# DNA Ministrings: Highly Safe and Effective Gene Delivery Vectors

**DOI:** 10.1038/mtna.2014.16

**Published:** 2014-06-03

**Authors:** Nafiseh Nafissi, Samih Alqawlaq, Eric A Lee, Marianna Foldvari, Paul A Spagnuolo, Roderick A Slavcev

**Affiliations:** 1 School of Pharmacy, University of Waterloo, Waterloo, Ontario, Canada; 2 Waterloo Institute of Nanotechnology, University of Waterloo, Waterloo, Ontario, Canada

**Keywords:** bacterial sequence-free DNA vectors, bacteriophage PY54 Tel/pal recombination system, DNA vaccines, gene therapy, genotoxicity, insertional oncogenesis, intercellular kinetics, MIDGE, minicircle DNA, ministring DNA, plasmid DNA vector, vector safety

## Abstract

Conventional plasmid DNA vectors play a significant role in gene therapy, but they also have considerable limitations: they can elicit adverse immune responses because of bacterial sequences they contain for maintenance and amplification in prokaryotes, their bioavailability is compromised because of their large molecular size, and they may be genotoxic. We constructed an *in vivo* platform to produce ministring DNA—mini linear covalently closed DNA vectors—that are devoid of unwanted bacterial sequences and encode only the gene(s) of interest and necessary eukaryotic expression elements. Transfection of rapidly and slowly dividing human cells with ministring DNA coding for enhanced green fluorescent protein resulted in significantly improved transfection, bioavailability, and cytoplasmic kinetics compared with parental plasmid precursors and isogenic circular covalently closed DNA counterparts. Ministring DNA that integrated into the genome of human cells caused chromosomal disruption and apoptotic death of possibly oncogenic vector integrants; thus, they may be safer than plasmid and circular DNA vectors.

## Introduction

Despite the great therapeutic promise of gene therapy (GT), both the safety and efficiency of DNA vectors need to be improved to realize their full clinical potential. Gene delivery systems can be viral or nonviral in design. Compared with viral DNA vectors, nonviral transgene delivery systems offer safer GT and vaccine design approaches, are less likely to elicit inflammatory and immune responses in hosts, have greater transgene capacity, and are easier to store. However, the effectiveness of nonviral GT delivery vectors is limited by low transgene expression levels, limiting their clinical application.^1^

Successful transgene delivery requires the DNA vector to enter the mammalian host nucleus and express the encoded gene(s). While simple in theory, in practice, several barriers must be overcome for translation to occur. The DNA vector encoding the gene of interest (GOI) must be immuno- and biocompatible to survive and traverse the extracellular environment. After crossing the cell membrane, the DNA vector must escape the endosome and make its way through the cytoplasm, ultimately to cross the nuclear membrane where it is transcribed. Until recently, most efforts have focused on modifying DNA delivery techniques by using improved synthetic carriers and physical methods. However, transfection efficiency (TE) can also be enhanced by modifying the composition and conformation of DNA vectors to improve their bioavailability, biocompatibility, durability, and safety.

Prokaryotic DNA sequences—such as CpG dinucleotide motifs, origins of replication, and antibiotic selectable markers—in plasmid DNA (pDNA) vectors lower their biocompatibility and safety, but these sequences are necessary to maintain and amplify pDNA vectors in bacterial hosts. In clinical studies, unmethylated CpG motifs induced inflammatory responses^2,3^ and necrosis- or apoptosis-mediated cell death in target cells, resulting in short-lived and compromised transgene expression.^4,5^ Furthermore, once they enter cells, the bacterial sequences of pDNA vectors are rapidly associated with histone indicators, packing the sequences into a dense heterochromatin structure. If these indicators spread into the adjacent GOI, the sequences can become inaccessible by transcription factors, leading to poor transgene expression or silencing of the eukaryotic promoter and GOI.^6^

Mini DNA vectors, including circular covalently closed (CCC) minicircles and linear covalently closed (LCC) MIDGE or MiLV are minimized GOI expression units devoid of bacterial backbone sequences that contain only the GOI and regulatory sequences.^7,8,9^ The MIDGE vector is an LCC dumbbell-shaped molecule, which is generated by digesting the parent plasmid vector *in vitro*, then ligating a hairpin cap to its open ends. MIDGE vectors have been used successfully as vaccine candidates with promising results *in vitro* and *in vivo*.^8,10,11,12,13^ In various *in vitro* and *in vivo* studies, MIDGE vectors showed great improvement with sustained and high transgene expression levels. Furthermore, by excluding the prokaryotic backbone and antibiotic-resistant genes of mini DNA vectors, MIDGE vectors have greatly enhanced immunocompatibility, reduce the risk of spreading antibiotic-resistant genes into body microbial flora, and have a transcriptionally active structure.^4,14,15^ Mini DNA vectors are also more likely to overcome obstacles during intracellular trafficking, improving their bioavailability compared with their larger parent pDNA counterparts.^7^ We previously reported a one-step *in vivo* prokaryotic platform to generate mini LCC DNA vectors processed to remove unnecessary plasmid sequences. This scalable system exploits the bacteriophage-derived Tel/*pal* recombination system that separates the LCC DNA conformation of the minimal cistron—intact with the GOI, promoter SV40, polyadenylation sequence, and four copies of the SV40 enhancer (SV40E)—from the unwanted plasmid backbone.^16,17^ The 72 bp SV40E sequence increases the import of pDNA into the nuclei of mammalian cells, thereby enhancing transgene expression.^18^ The SV40E sequence binds to at least 10 transcription factors that vicariously confer nuclear localization sequences to import the DNA-protein complex. SV40E acts as a scaffold for transcription factors and their bound importin proteins, causing the entire protein–DNA complex to be imported into the nucleus.

We hypothesized that mini LCC DNA vectors, which we call ministring DNA, should combine the benefits of minicircle DNA with the advantage of decreasing or even eliminating the risk of genotoxic insertions by causing apoptotic death of host cells that have inadvertently integrated the vectors, reducing the potential for proto-oncogene activation, tumor-suppressor gene deactivation, chromosomal DNA rearrangement or destabilization, and GOI silencing.

Our previous study showed chromosomal disruption and the lethality of a single crossover of an LCC pDNA vector in bacterial hosts.^16^ In this study, we compare the TE and intracellular distribution rates of ministring DNA vectors with those of isogenic minicircle DNA vectors and their parental plasmid precursors, and we investigate the cellular fate of human host cells when CCC and LCC pDNA are integrated into their chromosomes.

## Results

### Ministring DNA vectors exhibit higher transfection efficiencies

**Figure 1:**
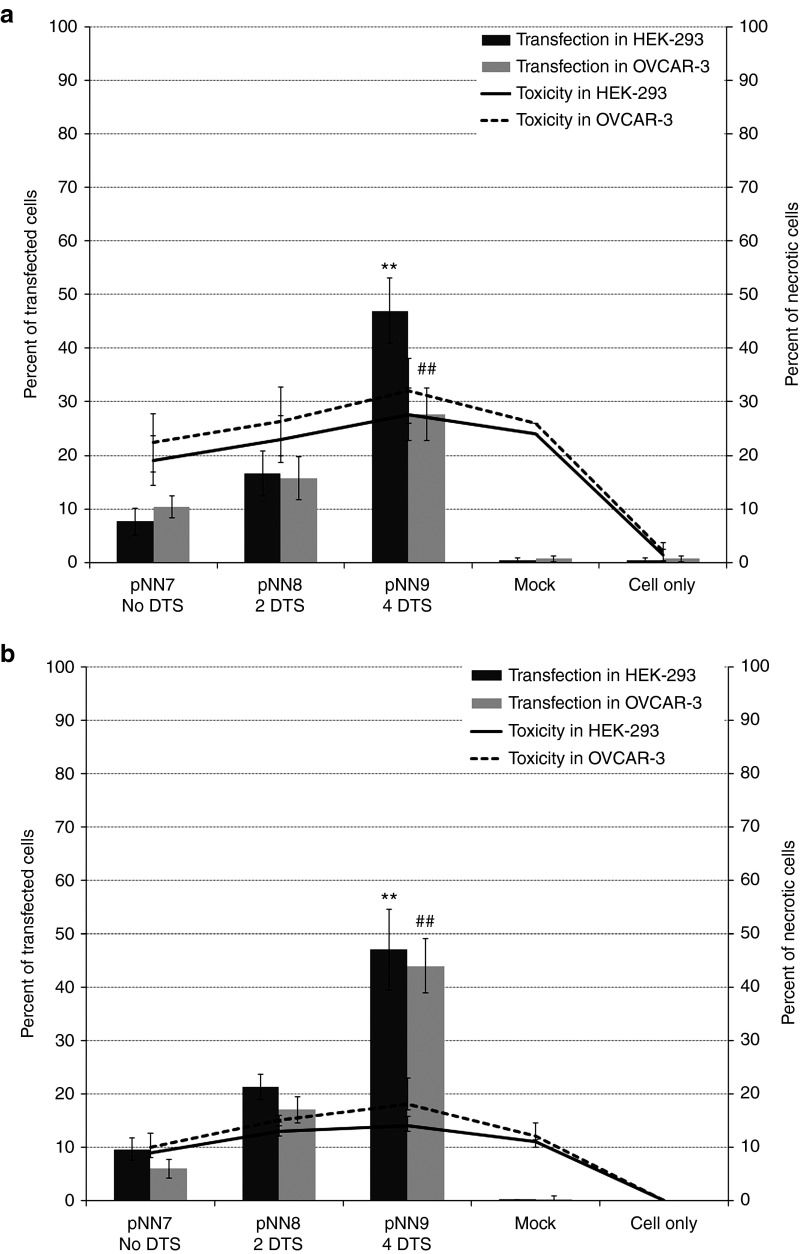
**Effect of SV40 enhancer sequence on transfection efficiency**. Lipoplexed/polyplexed DNA vectors were transfected into cancer and epithelial cells. Cells were collected 48 hours post-transfection and analyzed by FACS. Transfection efficiency (TE) was determined as the number of GFP-expressing cells divided by the total number of cells. Propidium iodide was added to measure transfection reagent cytotoxicity. In both HEK 293 and OVCAR-3 cells, (**a**) lipoplexed and (**b**) polyplexed pNN9 vectors (with 4 SV40E) show significantly higher TE compared with their pNN7 (with no SV40E) counterparts *(P* ≤ 0.001). DTS, DNA targeting sequences. FACS, fluorescence-activated cell sorting; GFP, green fluorescent protein.

**Figure 2:**
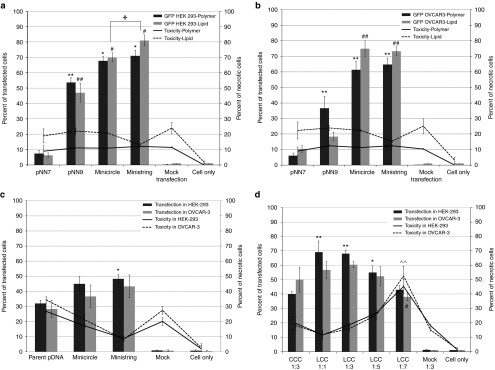
**Enhanced minivectors confer superior transfection efficiency in epithelial and cancer cells**. Enhanced pNN9 was processed into minicircle and ministring vectors by passing through Cre^+^ and Tel/TelN^+^ R-cells, respectively. DNA vectors were mixed by cationic polymer and cationic lipid carriers and transfected into cancer and epithelial cells. Cells were collected 48 hours post-transfection and analyzed by fluorescence-activated cell sorting for green fluorescent protein expression and cytotoxicity of cationic lipid/polymer carriers. Parent pNN9 is a 5.6 kb DNA molecule carrying two super sequence sites flanking the minimal eGFP cistron. Unlike pNN9, pNN7 does not possess SV40E, limiting its nuclear translocation efficiency particularly in nondividing cells. Transfection efficiency (TE) was measured as the number of eGFP-expressing cells divided by the total number of cells. Propidium iodide was added to measure synthetic carrier toxicity. (**a**) Slowly dividing epithelial cells and (**b**) rapidly dividing cancer cells transfected by 5 μg polyplexed or lipoplexed DNA vector. In both cell lines, mini DNA vectors show significantly higher TE (*P* ≤ 0.001). In epithelial HEK cells, ministring DNA showed significantly higher TE versus its minicircle counterpart (*P* < 0.05). (**c**) Cancer and epithelial cells transfected by 1 pmol lipoplexed DNA. In slowly dividing epithelial cells, ministring DNA showed significantly higher TE compared with parent pDNA using the same number of DNA molecules (*P* ≤ 0.05). (**d**) Cancer and epithelial cells transfected by 1 pmol of circular covalently closed (CCC) and linear covalently closed (LCC) pVGtelRL. pDNA was complexed with Lipofectamine 2000 (Invitrogen) at 1:3 ratio for conventional CCC plasmid and at different ratios for the isogenic LCC form of pVGtelRL (pDNA string), including 1:1, 1:3, 1:5, and 1:7. OVCAR-3 and HEK 293 cells were collected at 48 hours post-transfection and analyzed by FACS. In HEK 293 cells, the highest TE and lowest Lipofectamine-mediated toxicity were observed at 1:1 ratio of LCC pDNA to lipid transfection reagents (*P* ≤ 0.001). By increasing the volume of transfection reagents (cationic lipid carrier) to a 1:7 molar ratio, TE in OVCAR-3 cells was significantly lower than that seen under standard conditions (1:3) (*P* ≤ 0.05) and cytotoxicity was significantly higher (*P* ≤ 0.001).

We previously constructed a pGL2 (Promega, Madison, WI) vector derivative that expressed enhanced green fluorescent protein (*eGFP*) under the control of an SV40 promoter (pNN7). Next, we made pNN8 by adding a specially designed target sequence for Tel recombinase, which is flanked on both sides by the 76 bp SV40 enhancer sequence (SV40E), called super sequence (SS), upstream of the promoter.^16^ Another SS downstream of the polyA sequence was added to construct the enhanced pNN9 that carries four copies of SV40E (**Table 1**). The TE of pNN9 (four SV40E units) was significantly greater than that of pNN7 (no SV40E units) in both relatively slowly dividing epithelial (HEK 293) and rapidly dividing cancer (OVCAR-3) cells (**Figure 1**), demonstrating enhanced TE as the number of SV40E units is increased in the vector. Next, parental pDNA and derivative bacterial sequence-free LCC (ministring) and CCC (minicircle) DNA vectors carrying the *eGFP* expression cassette were produced using a one-step *in vivo* linearization and separation in our recombinant *Escherichia coli* (R-cell) system. Direct comparison with equal amounts (by weight) of ministring, minicircle, or conventional parent pDNA vectors in lipofected and polyfected HEK 293 and OVCAR-3 cells indicated by flow cytometry that excluding irrelevant bacterial segments of DNA vector, within a fixed amount of DNA, enhanced the copy numbers of the transgene expression units per cells. Isogenic minicircle and ministring-transfected cells conferred significantly higher TE than did the parental pDNA controls (*P* < 0.001) in either of cell lines (**Figure 2a**,**b**). Even, lipofection of HEK cells by ministring DNA led to statistically significant higher TE than minicircles (*P* < 0.05) with a 1.5-fold increase (**Figure 2a**). Similar results were observed in rapidly dividing OVCAR-3 cancer cells, and although the difference in TE between lipoplexed-enhanced minicircles and ministrings was not statistically significant (**Figure 2b**), a tendency of increased TE was observed for ministring DNA (*P* = 0.05).

In both cell types, lipoplexing increased the TE of mini DNA vectors more so than polyplexing did, while the opposite result was seen in the parent vector.

As a proof of higher efficiency of ministring DNA in transgene delivery, we lipofected both cell lines with equal molar ratios of ministring, minicircle, or conventional parent pDNA vectors. Higher TE was obtained by minicircle DNA compared to parent pDNA, although this increase was not statistically significant. However, lipofection by equal molar units of ministring DNA vectors conferred significantly higher TE than did the parental pDNA controls (*P* < 0.05) in epithelial cells (**Figure 2c**). These data indicate that at fixed DNA amounts, higher TE can be obtained with DNA ministrings compared to parent pDNA. Although at fixed DNA amounts, higher TE can be obtained with ministring versus isogenic minicircle, this improvement is likely not biologically relevant. If the number of copies interacting with the membrane is greater with ministring DNA vectors, it could explain why they enhance TE in comparison with the isogenic minicircle DNA, which is the same size (base pair numbers) and the same structure (nucleotide sequence) as ministring DNA (**Table 2**). We next tested the effect of DNA vector topology on lipid-mediated transfection and found that the cytotoxicity of cationic synthetic carriers complexed with DNA vectors was lower for ministring and minicircle DNA because these minivectors have a lower molecular weight than their parental precursors. Consequently, the concentration of lipid carrier can be reduced—especially so for ministring DNA vectors—as our optimization results indicate that LCC conformation requires a twofold lower cationic lipid carrier-to-DNA molar ratio than their CCC counterparts for the same size and sequence (**Figure 2d**).

### Enhanced ministring DNA vectors exhibit efficient cytoplasmic diffusion

The kinetics of cellular uptake, intracellular processing, and reporter gene expression were examined by confocal laser scanning microscopy in HEK 293 cells to determine the effect of DNA vector size and SV40E availability on interacellular diffusion and distribution of enhanced DNA minicircles and ministrings.

The parental precursor (pNN9) and ministring/minicircle derivatives were labeled before transfecting cancer and epithelial cells. They were then followed intracellularly at various times after transfection. The pVGtelRL plasmid was used as a positive control and a carrier-only vector as the negative/mock control. Vector intracellular kinetics revealed a remarkably faster nuclear uptake of pDNA vectors with four SV40E sequences compared with conventional plasmid, which had no SV40E sequences (data not shown).

**Figure 3:**
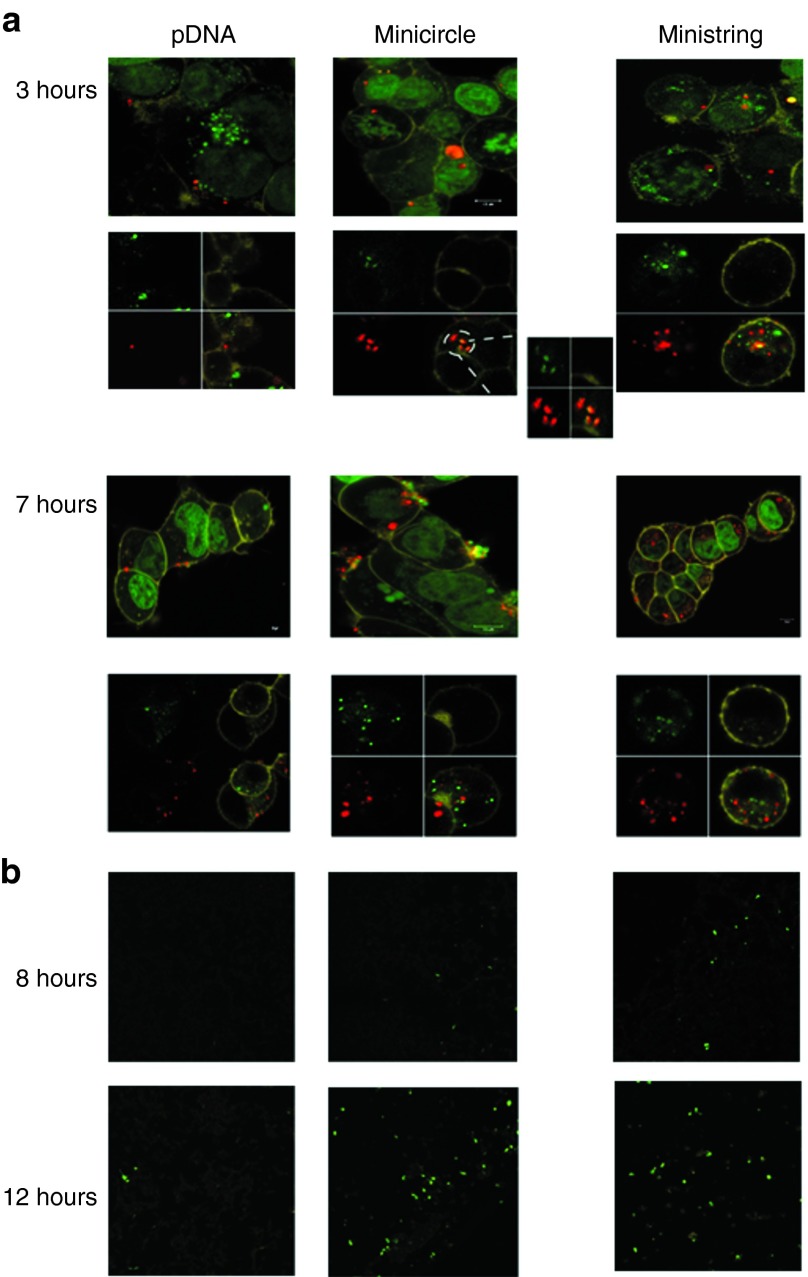
**Effect of pDNA size and topology on intracellular kinetics**. Confocal microscopic analysis of the uptake and distribution (panel **a**) and eGFP expression (panel **b**) of minicircle and ministring DNA in HEK 293 cells. Cells were imaged at 3 and 7 hours after dosing with the three different Cy5-labelled vectors complexed with Lipofectamine at 37 °C under two separate staining conditions: the first row of panel **a** 3- and 7-hour sections shows cells stained with SYTO 21 green nuclear stain and CellMask Orange cell membrane stain and the second row shows cells stained with LysoTracker green and CellMask Orange. The four pictures in the second rows represent signals in the three separate channels for LysoTracker green, CellMask orange, Cy5 and all combined (clockwise). Panel **b** shows eGFP expression for the three different vectors at 8 and 12 hours after incubation after removing the transfection complexes (transfection time was 6 hours).

Ministring DNA vectors were taken up by HEK 293 cells at a higher rate and extent than were isogenic minicircles and parent precursor pDNA (**Figure 3a**). At 3 hours, pDNA was seen mainly at and around the cell membrane, whereas minicircle and ministring DNA vectors were seen in the cytoplasm. Both minicircle and ministring complexes were encapsulated within late endosomes/lysosomes (**Figure 3a**, 3 hours second row). At 7 hours, uptake of pDNA was still low, whereas at the same time some minicircle complexes were seen near and within late endosomes/lysosomes, while ministring complexes had accumulated in significant amounts within late endosomes/lysosomes and in the cytoplasm, indicating that ministring complexes were released from lysosomes and located mainly at and around nuclei (**Figure 3a**, 7 hours second row). Expression of eGFP, assessed through green fluorescence, was observed earliest with ministring complexes, appearing first at 8 hours (**Figure 3**). At 12 hours, both minicircle and ministring complexes showed high levels of gene expression compared to parent pDNA.

### Integration frequency of LCC DNA vectors into the human genome was dramatically low

**Figure 4:**
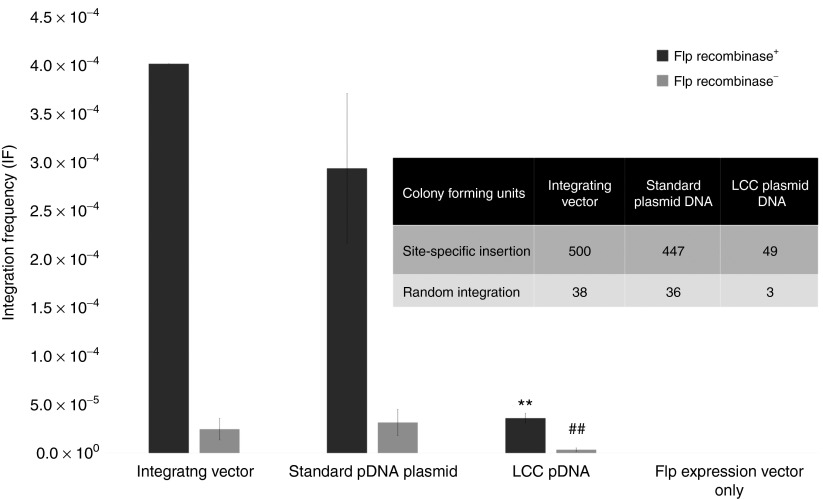
**Transgene integration frequencies of linear covalently closed (LCC) versus conventional circular covalently closed (CCC) pDNA into the human genome**. We directed site-specific insertion of LCC or CCC DNA constructs into the human (HEK 293) genome using the commercially available Flp-In System (Invitrogen) that exploits the Flp-*FRT-*mediated recombination system. Efficacy of LCC and standard pDNA vector chromosomal integration were compared by Flp-*FTR*-mediated site-specific integration to verify and compare the safety profile of LCC versus standard pDNA vectors. Flp-In 293 HEK cells (Invitrogen) carrying the attachment site (*att*), were transfected by the Flp-In kit plasmids pcDNA5/FRT and pcDNA5/FRT/CAT that possess the Flp target site *FRT* and the indicator chloramphenicol acetyl tranferase (*CAT*). The super sequence was cloned into each plasmid and passed through Tel^+^ R-cells to generate plasmids with LCC topology. Transfection was performed in the presence or absence of Flp-recombinase expression vector, pOG44, for site-specific single crossover or random integration, respectively. Transfected cells were selected with hygromycin for 3 weeks, and resistant colonies were counted as a measure of colony forming units. Mean ± SEM values are shown (*n* = 3–5). The Hyg^R^, Zeo^S^, and B-Gal^−^ cells represent the result of Flp-mediated site-specific insertion of the gene of interest. In contrast, Hyg^R^, Zeo^R^, and B-Gal^**+**^ cells represent low-level illegitimate integration events. The mean was calculated from a minimum of three trials and with different plasmids. Integration frequency (IF) is expressed as the fraction of integrant cells to total transfected cells. IF of LCC was significantly lower than its CCC counterpart and control plasmid with no super sequence, (*P* < 0.001).

We examined the rate of stably transfecting HEK 293 cells using standard or LCC pDNA integrating vectors using the commercially available Flp-In System (Invitrogen) that exploits the Flp-*FRT* high-efficiency recombination system. LCC or standard pDNA integration vectors were lipofected in with the presence or absence of Flp recombinase expression vector to direct site-specific or random insertion of the GOI into target cell genome, respectively. After antibiotic selection, viable integrated clones were isolated and expanded 3 weeks post-transfection/selection. The number of colony forming units and integration frequency (IF) of cells that were lipofected by LCC pDNA was more than 150-fold lower than that of isogenic standard pDNA counterpart vector integrants (**Table 3** and **Figure 4**).

### Integration of LCC DNA into the host chromosome results in chromosomal disruption and cell death

**Figure 5:**
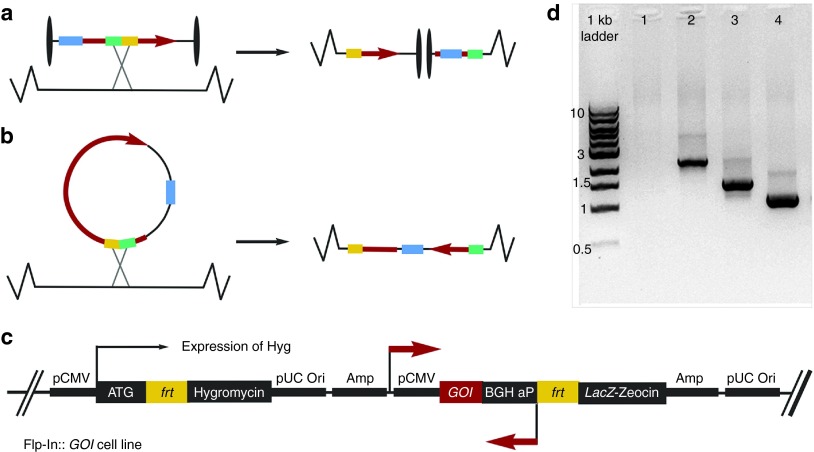
**Linear covalently closed (LCC) integration results in chromosomal disruption at the site of vector insertion**. Schematic representations of linear and circular vector integration events.^16^ A mini DNA vector that undergoes a single recombination event with the host chromosome is rare because all elements have been removed except the cistron containing the gene of interest expression cassette and the flanking super sequence sites. (**a**) LCC DNA vector integration would result in a chromosomal break at the integration site, whereby the chromosome cannot replicate or segregate and the integrated mammalian cell cannot divide because its centromere has separated from the telomere. (**b**) A minicircle vector can integrate into a nonessential region of the host chromosome without breaking the chromosome, thus the cell can continue to divide. (**c**) Schematic representation of the primers located on the Flp-In 293 genomic DNA and used to amplify the site of integration. These primers are specific for the Flp-In kit that amplifies over the region of vector insertion and the expected size for the intact host cell with no insertion or with the positive CAT insertion are 240 or 1,058 bp, respectively. (**d**) LCC integration events were tested by PCR at site of integration. Equal numbers of colonies of different size were randomly picked from each experiment. CMV and BGH primers were used to amplify genomic DNA extracted from the Hyg^R^, Zeo^S^, and B-Gal^−^ cells. From left to right: 1 kb ladder; L1: Flp-In::LCC CAT (linearized plasmid control for disrupted sequence yielding no bands); L2: Flp-In::*tel* (240 bp + 1,900 bp); L3: Flp-In::*SS*-CAT (342 bp + 1,058 bp); L4: Flp-In::CAT (positive control) (1,058 bp). This analysis was repeated with identical findings.

**Figure 6:**
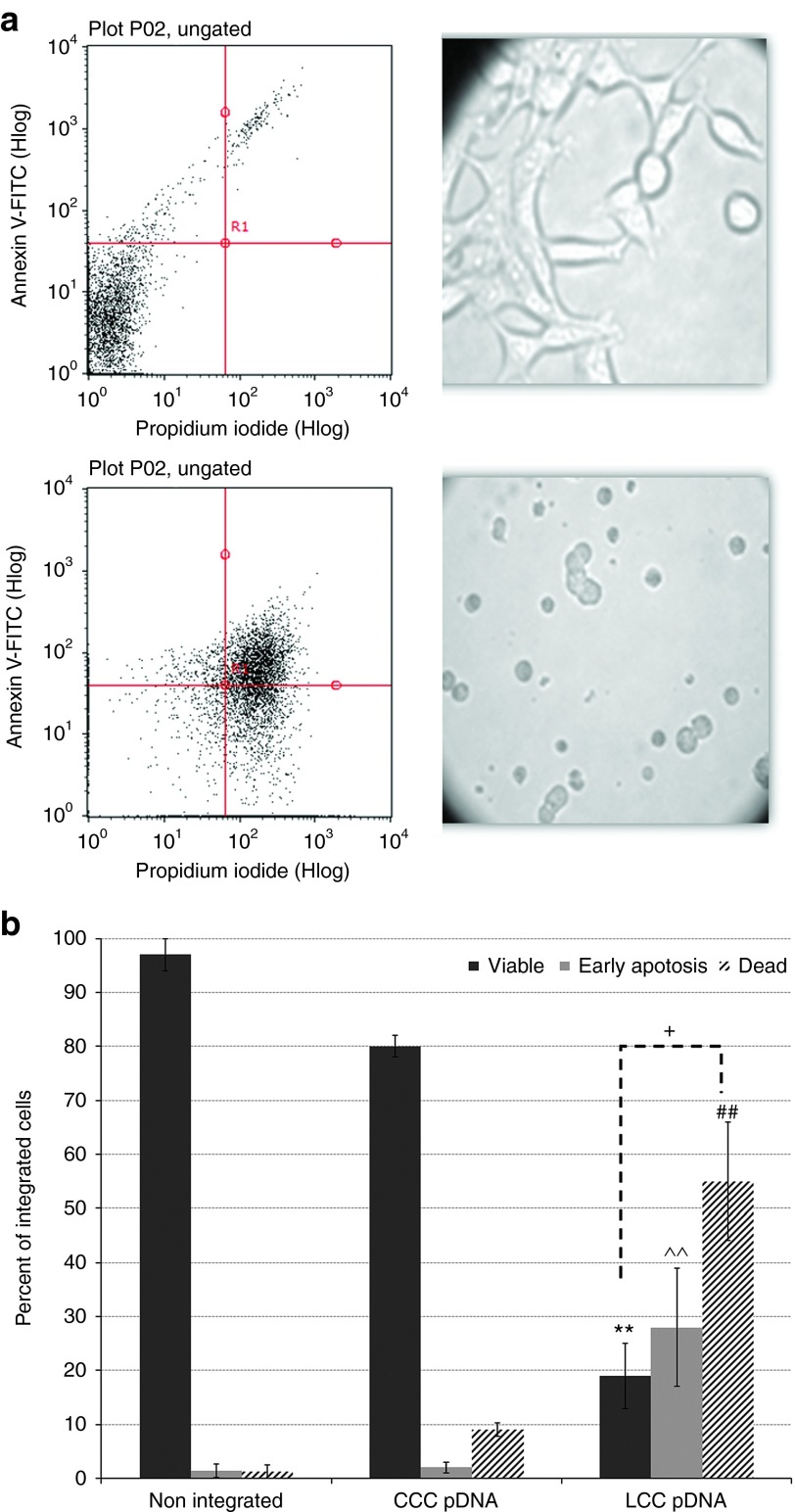
**Linear covalently closed (LCC) single crossover integration into human cell induces apoptotic cell death**. Any surviving LCC and circular covalently closed (CCC) pDNA-mediated Hyg^R^, Zeo^S^, B-Gal^−^ cells were isolated and expanded in six-well culture plates. To determine fate of these cells after site-specific insertion of the LCC and standard pDNA vectors, expanded clones were collected and pooled at 3, 5, and 7 weeks post-transfection. To determine whether cells that had integrated the LCC vector now possessed, as hypothesized in Figure 5b, a chromosomal disruption at the site of vector integration, 106 cells of each group were stained by Annexin V-FITC, and propidium iodide (PI) and results were analyzed by flow cytometry. Measurements were normalized against controls: (i) unstained nonintegrated cells, (ii) nonintegrated cells stained with only Annexin V, and (iii) nonintegrated cells stained with only PI. (**a**) Two-channel reading of flow cytometry results aligned with cell morphology of CCC (upper) and LCC (bottom) integrants. (**b**) Morphology of integrated cells. Graphs represent percentage of healthy, apoptotic, or necrotic cells. Mean of a minimum of three trials. LCC integrants show significantly lower viability and higher apoptotic and necrotic index compared with the CCC integrants (*P* < 0.001). The number of dead cells within the LCC integrant group is significantly higher than healthy cells (*P* < 0.05). In general, the LCC pDNA-mediated integrants were found to be dramatically smaller in size and arrested in cell division compared to a completely normal morphology and growth rate seen with standard pDNA vector-mediated integrants.

We examined the safety profile of ministring DNA vectors and hypothesized that although the integration of DNA vectors into the host genome would occur rarely, integration of an LCC DNA vector into a human host chromosome would disrupt it at the site of integration, reducing the stability of the genome, either killing the aberrant integrant cell or targeting it quickly for apoptosis (**Figure 5a**). In contrast, because of its circular topology, we hypothesized that the integration of a CCC vector would not result in such disruption (**Figure 5b**). To test these hypotheses, we forced site-specific insertions of the LCC or CCC pDNA constructs into the genome of Flp-In cells (Invitrogen) that have been used conventionally in studies of sequence-specific insertion of the GOI. Cells that integrated the CCC or LCC pDNA vector were collected and tested at 3–5 weeks post-transfection/integration. To determine whether cells that had integrated the LCC or CCC vector had disrupted chromosomes at the integration site, all LCC and CCC integrants were collected and assayed for chromosome integrity by PCR amplification over the integration site (**Figure 5c**). The LCC integrant DNA pool demonstrated chromosomal disruption as shown by the inability to amplify DNA over the integration site, whereas CCC integrants demonstrated the expected presence and size of integrated DNA inserts (**Figure 5d**). Surviving LCC integrant cell colonies were isolated and found to be much smaller and in arrested cell division compared with the normal morphology and growth rate seen among CCC cell integrants (**Figure 6a**). LCC integrants were tightly restricted for growth and division, and we were not able to collect sufficient cells after the third passage (7 weeks post-transfection) to further conduct more experiments. We also noted that as the passages increased, the viability of LCC integrants, but not the CCC counterparts, were dramatically compromised.

We next sought to determine whether aberrant LCC integrant cells were viable or at various stages of cell death. To assess cell fate after LCC or CCC DNA vector integration, Annexin V-FITC and propidium iodide (PI) staining and flow cytometry were employed to distinguish viable from early apoptotic cells (Annexin V positive/PI negative) or late apoptotic/necrotic cells (Annexin V and PI positive). More than 50% of LCC integrants were at a stage of cell death compared with a significantly healthier population of CCC integrant cells (**Figure 6**).

## Discussion

GT and modern vaccination require safe and efficient expression vectors. Nonviral DNA-based gene delivery offers attractive opportunities for molecular medicine, but the TE of pDNA is low, likely because of inefficient DNA uptake by cells, poor cytoplasmic diffusion and nuclear uptake rates, and low levels of transgene expression. Design and optimization of highly efficient synthetic carriers and generation of compact and bacterial sequence-depleted DNA vectors can circumvent these limitations dramatically. We combined an enhanced mini DNA vector—ministring DNA—with an efficient transfection method, using cationic lipid and polymer carriers. Ministring DNA represents the next generation of plasmid-derived DNA delivery systems. These vectors possess LCC ends, minimal transgene expression cassette elements devoid of prokaryotic sequences, and DNA targeting sequences (DTS) at both ends. Such processing significantly reduces the size of the vector. Previously, we described the exploitation of recombination systems encoded by bacteriophages PY54 and N15, to generate enhanced isogenic DNA ministring or minicircle vectors using a one-step *in vivo* production platform.^16,17^ In this study, we compared the TE and cytoplasmic kinetics of ministring DNA with those of parent plasmid and isogenic minicircle DNA vectors and further investigated their safety by determining the site-specific and random integration efficiency and fate of cells after a forced Flp recombinase-mediated sequence-specific integration of LCC and conventional plasmid DNA vectors into the genome of human host cells.

Standard pDNA vectors have a number of shortcomings, including containing bacterial sequences and antibiotic-resistant genes that can trigger immune responses in mammalian hosts, large molecular size, and the potential to cause mutations and genotoxic effects near the insertion site. More recent DNA vectors have been optimized to contain few minimal immunogenic components while improving TE. The absence of bacterial DNA sequences results in more robust and persistent transgene expression *in vivo*.^19^ Mini DNA vectors can have either a CCC or LCC conformation and their production can be accomplished by using phage recombination systems.^16,17,20^ Mini DNA vectors are a promising alternative to conventional plasmids. Because of their smaller size and lack of immunostimulatory bacterial sequences, these vectors show amended biosafety, better biological/immunological compatibility, improved gene transfer, potentially greater bioavailability, and higher cytoplasmic diffusion rates.^5,15^

Common nonviral DNA transfection procedures use chemical methods, including lipid- and polymer-mediated DNA transfer, and physical methods, including electroporation and microinjection. The majority of previous investigations have focused on assessing the efficiency of transgene expression by testing the transgene and its protein product expression level over the time, but little is known about the kinetics of transgenic DNA intracellular diffusion and its localization to the nuclear membrane of the target cell. Previous studies on different types of linear closed vectors, such as MIDGE and MiLV, indicated a higher and more sustained (durable) expression levels of the transgene and its protein *in vitro* and *in vivo* using equal weight ratio compared to the parent plasmid vector, even though the difference is very tissue specific.^7,8,13^ Furthermore, most conventional methods for monitoring gene transfer assess protein expression of reporter genes, such as *luc* and *gfp*, from 24 to 48 hours to few month post-transgene delivery into the target site. However, there is a lack in our understanding on early or intermediate transfection events. Here, we describe a method to monitor intracellular diffusion of modified or unmodified DNA vectors within a few hours post-transfection.

Recent results have shown that DNA vector–cell membrane interaction is necessary but not sufficient for transgene expression, suggesting that a minimum number of DNA copies need to interact with the permeabilized cell membrane to promote vector internalization and transgene expression. If the number of copies interacting with the membrane is greater with ministring DNA vectors, it could explain why they enhance TE in comparison to the isogenic minicircle DNA, which is of the same size (base pair numbers) and the same structure (nucleotide sequence) as the ministring DNA. Translocation of DNA across the nuclear membrane is the next crucial step for gene expression. The intracellular kinetics of ministring DNA vectors is inversely proportional to their molecular weight and that, along with their linear topology, may help promote gene expression. In slowly dividing cells, transgene delivery by ministring DNA vectors demonstrated superior cellular uptake, TE, and GOI expression than transgene delivery by both minicircles and the parental plasmid precursor. Compared with their CCC counterparts, DNA in LCC MIDGE vectors improved gene expression level up to 17-fold in specific tissues.^8^ Both LCC and CCC mini DNA vectors devoid of bacteria sequences demonstrated improved cytoplasmic diffusion because of their smaller size, but ministring DNA had preferable kinetics relative to its isogenic minicircle, suggesting that DNA conformation is important. Furthermore, incorporating DTS was shown to facilitate vector nuclear localization in human embryonic kidney cells, which relative to ovarian cancer cells are slowly dividing. The four SV40E sequences in CCC and LCC vectors likely contribute to enhance TE exhibited by both mini DNA and parent plasmid vectors in comparison with the conventional plasmid carrying no SV40E in both cell lines (**Figure 1**). These findings agree with previous reports on the impact of DTS on transgene expression levels.^18,21^ We showed that in addition to the size of DNA vectors, DNA topology and conformation also influence cytoplasmic distribution. Bacterial sequence–free ministring DNA showed higher TE with both cationic lipid- and polymer-mediated delivery, which, as in minicircles, can be attributed to their smaller size, better cytoplasmic diffusion rates, and improved DTS-mediated nuclear uptake relative to the parent plasmid. We also showed that there is greater intensity of fluorescence in HEK cells lipofected by an equal molar ratio of eGFP ministrings versus the isogenic minicircle DNA vectors and parent plasmid counterparts (**Figure 2c**), which again can be attributed to quantitatively higher ministring DNA copy numbers taken up, diffused, translocated, and expressed per cell. Live cell confocal imaging to analyze the cellular uptake, lysosome escape, and intracellular diffusion of the isogenic eGFP expression ministring and minicircle DNA vectors also visually supported enhanced uptake, lysosomal escape, and higher ministrings copy numbers per transfected cell, as well as improved cytoplasmic diffusion and earlier transgene expression (**Figure 3**). However, the biological relevance of superior efficacy of ministring DNA is questionable and requires further in-depth *in vivo* testing in various tissues and via a variety of delivery methods. We are currently investigating whether additional SV40 E sequences will continue to improve TE in nondividing cells, as TE has demonstrated to improve when DTS are added to the vector, and to what extent, heightened expression is conferred by ministrings across a wide variety of tissues.

In order to determine whether the safety of LCC vectors in human cells is amenable to other DNA vectors, we compared the integration rate of LCC versus standard CCC pDNA. Efficient Flp-mediated vector integration of conventional CCC but not LCC pDNA indicates the added safety of LCC DNA vectors over standard plasmid and other circular DNA vectors. While bacterial sequence-free mini DNA vectors offer improved efficacy, LCC vectors such as ministrings lead to a lower number of integration events and cause rearrangements at insertion sites. We also investigated the natural integration event of vectors in the absence of Flp recombinase and while the resultant low efficiency of integration and low integrant number makes it difficult to draw conclusions with any degree of certainty, we did again find that LCC DNA vectors initially integrated at much lower frequency and the integrant colony forming units were significantly lower than the number of standard plasmid integrants (**Figure 4** and **Table 3**). Circular DNA vectors can compromise safety because they integrate freely into the host DNA, whereas if LCC DNA vectors are integrated into host DNA, these cells become targeted for death. As expected, we found that when LCC DNA vectors are integrated into host chromosomal DNA they disrupt the chromosome (**Figure 5**) by separating its centromere from the telomere. Such genomically unstable integrant cells have arrested growth and are targeted for apoptosis. The natural elimination of such cells prevents propagation of potentially genotoxic integrants in the transfected cell population, thereby providing a safer option for DNA vector-mediated transgene delivery.

Enhanced bacterial sequence–free linear DNA vectors with covalently closed ends, what we have termed DNA ministrings, are a new generation of bacterial sequence-depleted transgene delivery vehicles. They represent robust nonviral gene delivery vectors and may have various applications in molecular medicine, industry, and biotechnology. We previously generated these vectors using a one-step enzymatic reaction in our novel *E. coli* cells. In addition to expedited cytoplasmic diffusion and improved TE, ministring DNA vectors have a superior safety profile because they have lower immunogenicity and inferior tendency of integration into the host genome, and if they are integrated, integrants are naturally eliminated by apoptotic-mediated cell death.

## Materials and methods

*Strains and plasmids. E. coli* K-12 strains were used to generate recombinant cell constructs, and JM109 bacterial hosts were used for plasmid maintenance and amplification. Bacterial strains used, and plasmids constructed in this study are shown in **Table 1**.

*Vectors and cell lines.* Plasmid pcDNA5/FRT and pcDNA5/FRT/CAT integrating vectors, and pOG44 integrase-expressing vector were obtained from Invitrogen (Carlsbad, CA); pGL2 expression vector was obtained from Promega. Restriction enzymes and DNA-modifying enzymes were obtained from New England Biolabs (Ipswich, MA). HEK 293 and OVCAR-3 cells were obtained from Invitrogen and grown in the respective complete media: high-glucose Dulbecco's modified Eagle medium (DMEM) + sodium pyruvate + GlutaMAX supplemented with 10% fetal bovine serum, 100 µg/ml streptomycin, and 100 IU/ml penicillin, and RPMI+ GlutaMAX supplemented with 20% fetal bovine serum, 100 µg/ml streptomycin, and 100 IU/ml penicillin. Flp-In 293 cells were obtained from Invitrogen and grown in DMEM complete media supplemented with 100 µg/ml zeocine. The GFP-expressing pVGtelRL vector (a gift from Dr Jochen Heinrich, Germany) was used as an internal control for TE. All cell culture reagents were provided by Life Technologies (Carlsbad, CA), cell culture equipment from VWR (Radnor, PA) and Fisher (Waltham, MA), and chemical reagents from Fisher and Sigma-Aldrich (St Louis, MO).

*Construction and characterization of ministring DNA.* The multipurpose target site, named SS, was designed to carry two flanking 78 bp SV40 enhancer sequences to facilitate nuclear translocation and enhance TE. It was moved to pGL2 vectors to generate plasmid vectors pNN7, pNN8, and pNN9.^16^ New constructs were tested and confirmed by colony PCR and analytical digestion. The CCC pVGtelRL/pNN7 and pNN9 (*eGFP*-expressing vectors with no and with four DTS, respectively) were converted to LCC pDNA, DNA minicircles, or DNA ministrings by passing 1 µg of the conventional corresponding plasmids through R-cells. R-cells were then grown on LB + Ap (100 µg/ml) to *A*_600_ = 0.8 at 30 °C with aeration. To induce recombinase expression and plasmid conformational conversion, cells were collected at late logarithmic phase of bacterial growth by centrifuge at 12,000 RPM for 2 minutes. The media was then removed and transformed R-cells were transferred to a new culture flask with media preheated to 42 °C for heat shocking and to induce recombinase expression at 42 °C for 30 minutes at late logarithmic phase of bacterial growth, before being transferred to 30 °C overnight. Cells were then harvested and plasmid was extracted (endotoxin-free plasmid isolation maxi kit; Omega, VWR). DNA vector topology was assayed by agarose gel electrophoresis, ethidium bromide staining, and analytical digestion. Standard recombinant DNA techniques were performed as described in ref. 22.

*TE assay of ministring DNA vector.* Cationic polymer transfection reagents (jetPRIME) were obtained from VWR and cationic lipid transfection reagents (Lipofectamine 2000, Lipofectamine LTX, and Plus reagents) from Invitrogen. To transfect cells using these reagents, 0.5–1 × 10^6^ OVCAR-3 and 1–2 × 10^6^ HEK 293 cells were seeded into six-well culture plates 24 hours before transfection in complete media without antibiotic. One to two picomol DNA vectors (**Table 2**) were mixed by lipid- or polymer-based carriers for each well. One hour before transfection, the culture medium was replaced with serum-free medium. DNA and the transfection reagent were then mixed with 0.5-ml serum-free OptiMEM culture medium in separate tubes and incubated for 10 minutes at room temperature. Cationic complex jetPRIME or lipofectamine solution was added to the DNA solution, mixed by vortexing, and incubated for an additional 30 minutes at room temperature. Medium culture was removed from the plate and the mixture of transfection-reagent and DNA was added dropwise. The culture was centrifuged for 5 minutes at 200 RPM at room temperature. Plates were incubated at 37 °C, and after 2 hours, six-well plates were filled up to 2 ml on complete medium without antibiotic. TE was controlled after 48 hours. Ratios of DNA and transfection reagent were optimized for the two cell lines and for LCC pDNA. Different combinations of transfection reagent (1–10 µl cationic complex, corresponding to 1–2 pmol pDNA) were tested.

*Flow cytometry.* TE was determined 48 hours after transfection by flow cytometry using an Epics XL BD FACSVantage SE, BD Bioscientific (Department of Biology, University of Waterloo). Cells were trypsinized 48 hours after transfection, washed with PBS, and counted. Data were collected from 10^6^ cells. Ten microliters of PI, 20 mg/ml Sigma-Aldrich (St Louis, MO) were added to measure toxicity after transfection by detecting dead cells. Cells with no transfection and cells transfected only with transfection reagent served as PI and mock transfection controls, respectively. Cells transfected by 1 pmol of pVGtelRL served as the GFP control. Indicator GFP expression levels were calculated by multiplying the mean relative fluorescence values of transfected cells by the percentage of transfected cells. This parameter is considered to be directly proportional to the total amount of produced transgene product.

Integration-mediated apoptosis was detected using the AnnexinV-FITC (ANN)/PI staining assay (BD Biosciences, Franklin Lakes, NJ), as previously described.^23^ ANN binds to plasma membrane–associated phosphatidylserine on the cell surface, and PI intercalates with DNA only by entering the cell through a broken plasma membrane. Therefore, flow cytometry readings of ANN^−^/PI^−^, ANN^+^/PI^−^, and ANN^+^/PI^+^ indicate that a cell is viable, early apoptotic and dead, respectively. Flow cytometry was performed using a Guava easyCyte 8HT flow cytometer (Millipore, Billerica, MA).

*Statistical analysis.* Data were analyzed by one-way analysis of variance; *P* < 0.05 was considered statistically significant. Each error bar represents the standard deviation of a minimum of three separate experiments.

*DNA vector labeling and real-time assay of intracellular kinetics of ministring DNA.* Modified pGL2 pDNA-expressing GFP (pNN7) with no DTS and (pNN9) flanking two SS and four DTS, ministring and minicircle DNA vectors carrying the *eGFP* expressing cassette were labeled with the *Label* IT Tracker Reagent Cy5 (excitation wavelength 649 nm and emission wavelength 670 nm from Mirus, Brampton, Ontario, Canada) at a molarity ratio Cy5:DNA of 0.5:1 and incubated for 3 hours at 37 °C. Unreacted Cy5 was removed from the labeled DNA by ethanol precipitation. Labeled DNA was resuspended in 10-µl sterile nuclease–free water. Concentration of the purified, labeled DNA was measured by a NanoDrop spectrophotometer, and the integrity of labeled DNA was quantified by agarose gel electrophoresis before and after labeling with and without ethidium bromide staining to confirm the direct and nondestructive nature of the labeling reaction. Labeled DNA was protected from light. OVCAR-3 or HEK 293 cells were seeded on noncoated 24-well glass bottom plates (Greiner bio-one, GmbH, Germany), at a density of 10^4^ cells per well in complete media without antibiotic such that their confluency was ~70% at the time of imaging. These cells were transfected with 0.5 µg of Cy5-labeled DNA vectors pNN7, pNN9, ministring and minicircle *eGFP* expression DNA vector, and pVGtelRL as an internal GFP expression control using lipoplexed DNA that was prepared by diluting Lipofectamine 2000 and labeled DNA 3:1 in serum-free OptiMem media, incubated for 30 minutes at room temperature before transfection. Images were taken with a Zeiss LSM 710 laser scanning confocal system with a ×63 oil-immersion objective at various times: 3, 5, and 7 hours after transfection. Cells were costained with a combination of SYTO 21 green nuclear stain and CellMask Orange cell membrane stain or with the combination of LysoTracker green and CellMask Orange. The lysosomal stain, LysoTracker green (excitation: 504 nm, emission: 511 nm; Invitrogen, Grand Island, NY) was applied at a final concentration of 40 nmol/l for 20 minutes at 37 °C. Nuclear localization was assessed separately using the nuclear stain, SYTO 21 (excitation: 494, emission: 517 nm; Invitrogen) at a final concentration of 2 µmol/l for 5 minutes at room temperature. Both stains were visualized using an argon 488 nm laser. Each stain was applied conjointly with the cell membrane stain, CellMask Orange (excitation: 554, emission: 567 nm; Invitrogen), which was applied at a final concentration of 5 µg/ml for 5 minutes at room temperature, and visualized using a 560 nm argon laser. GFP expression was evaluated in separate plates without cellular stains at 8, 9, and 12 hours after transfection, using cell CellMask Orange as a counterstain, using a 488 nm argon laser.

*Construction and characterization of LCC-integrating pDNA vectors.* To construct modified integrating vectors, the SS fragment was moved from pUC57 into the multiple cloning site of pBRINT (Cm^R^) plasmid by *Bam*HI and *Eco*RI to generate pNN12, and moved from pNN12 by *Bam*HI and *Xho*I into the multiple cloning site of pcDNA5/FRT and pcDNA5/FRT/CAT vectors (Invitrogen) to produce pNN13 and pNN14, respectively (**Table 1**). New constructs were tested and confirmed by colony PCR and analytical digestion. One microgram of supercoiled CCC pNN13 and pNN14 constructs were electroporated into W3NN R-cells (*tel*^*+*^) and grown on LB + Ap (100 µg/ml) to *A*_600_ = 0.8 at 30 °C with aeration. To induce recombinase expression and plasmid conformational conversion, transformed cells were heat shocked at 42 °C for 60 minutes to induce the Tel recombinase expression at late logarithmic phase of bacterial growth, before being transferred to 30 °C overnight. Cells were then harvested and the plasmid was extracted (Omega maxi plasmid extraction kit; VWR). LCC pDNA topology was assayed by agarose gel electrophoresis, ethidium bromide staining, and analytical digestion.

*Assessing the chromosomal IF of LCC DNA versus conventional CCC DNA vector.* Flp-In 293 cells (5 × 10^5^ cells/well) were seeded in a six-well plate with 2 ml complete DMEM media without antibiotic, such that their confluency was ~80% at the time of transfection. Cells were cotransfected by 0.3 µg of CCC and LCC forms of pNN13 (pcDNA5/FRT+SS) and CCC and LCC forms of pNN14 (pcDNA5/FRT/CAT+SS) with a 9:1 (integrase:pDNA) molarity ratio of Flp recombinase expression vector pOG44:integration pDNA vectors. pDNA vectors were mixed by Lipofectamine LTX and Plus reagents to the 1:3 DNA:lipid and 1:0.5 DNA:plus helper lipid, and incubated for 30 minutes at room temperature to produce lipoplexed DNA in serum-free OptiMem media. We included the following controls: (i) untransfected cells as a negative control, (ii) pcDNA5/FRT/CAT integration vector as a positive control, (iii) pCDNA5/FRT/GOI transfected without pOG44 to see when the selection is complete and measure random IF, and (iv) mock transfection by Flp recombinase expression vector pOG44 only as negative control. Fourty-eight hours post-transfection, transfected cells were collected and expanded into 100-mm plates containing complete DMEM media with no zeocin, in such a way that they were not more than 25% confluent. After cells were attached to the new plates, hygromycin was added to a final concentration of 200 µg/ml. Selection and expansion of stably integrated cells were performed in the presence of hygromycin for 3–7 weeks post-transfection. Three weeks post-transfection, the site-specific integrated cells were tested for lack of β-galactosidase activity after B-gal staining kit protocol from Invitrogen with no deviation. CAT expression level was measured following CAT ELISA protocol (Roche Canada, Mississauga, Ontario, Canada), with no deviation, to test the positive control integration vector efficacy. Zeocin sensitivity test was performed by growing cells in a gradient concentration of zeocin: 0, 25, 50, 75, and 100 µg/ml. To further verify that the random integration was occurring, hygromycin and zeocin selection was performed for 5 weeks. Resistant clones were counted to record the colony forming units and this number relative to the total number of transfected cells was recorded as IF.

*Assessing fate of LCC DNA vector chromosomal integrant cells.* To further verify that the site-specific recombination was occurring at the *FRT* site and to test the fate of integrated cells, at 3–5 weeks post-transfection, genomic DNA isolated from Hyg^R^, Zeo^S^, B-Gal^−^ Flp-In:: *GOI* cells was subjected to PCR with pCMV- F and BGH-R specific primers that are located on the host cell chromosome adjacent to the site if insertion (*att*) and would amplify only at the integration site. New cells were expanded as Flp-In::*cat*, Flp-In:: *SS-cat*, and Flp-In:: *SS*.

*Viability assay of LCC DNA integration.* The Hyg^R^, Zeo^S^, B-Gal^−^ CCC and LCC integrant cells (10^6^) were collected at 3, 5, and 7 weeks post-transfection and subjected to apoptosis assay. Trypsinized cells were washed two times by cold PBS and 250 µl of the Annexin V, and PI solution were added and incubated in the dark for 15 minutes at room temperature and then measured by flow cytometry. Data were normalized by three controls: unstained nonintegrated cells, nonintegrated cells stained with only Annexin V, and nonintegrated cells stained with only PI (ApoAlert Annexin V Apoptosis kit from Clontech, Mountain View, CA).

## Figures and Tables

**Table 1:**
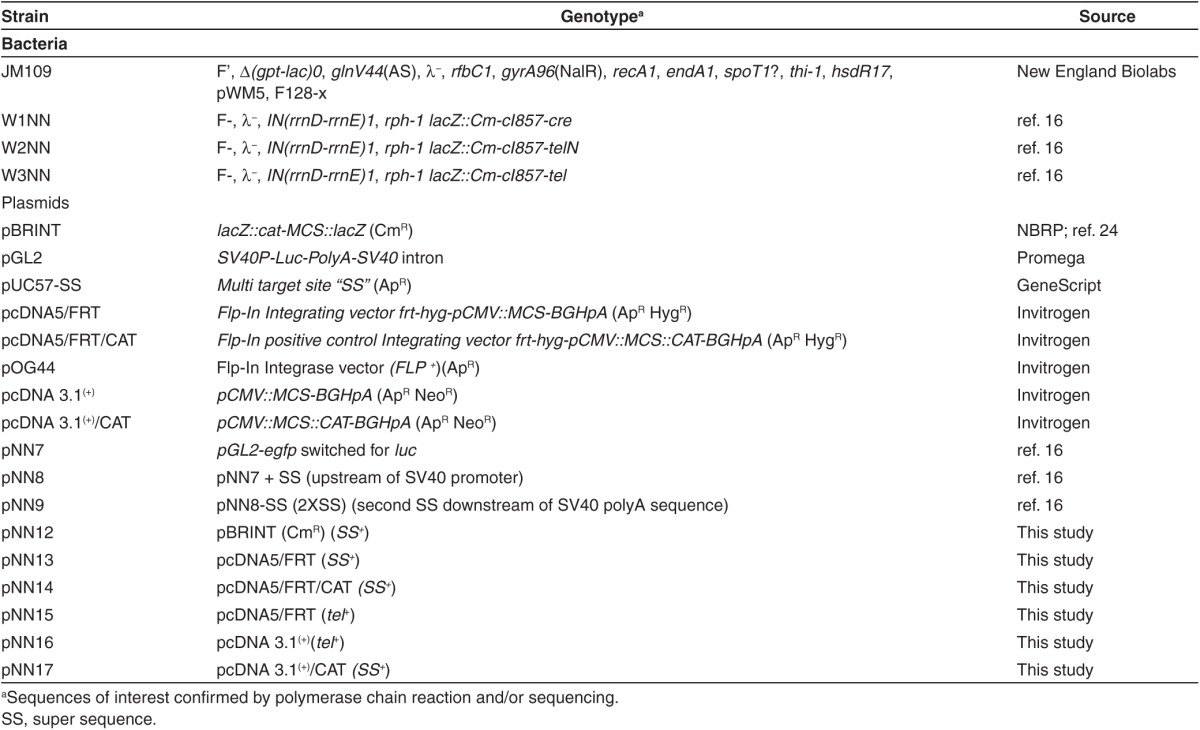
Strains and plasmids

**Table 2:**
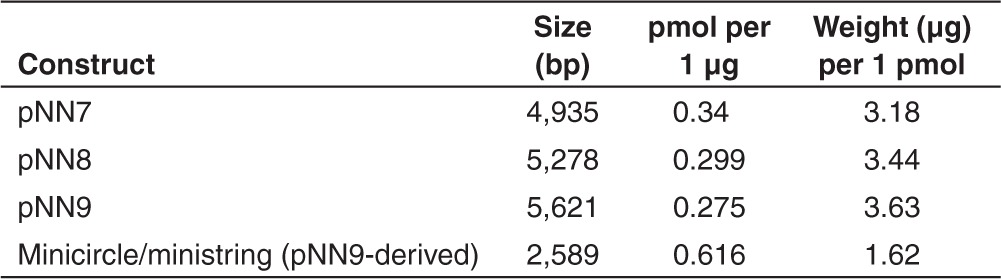
DNA vectors used to measure transfection efficiencies in epithelial and cancer cells

**Table 3:**
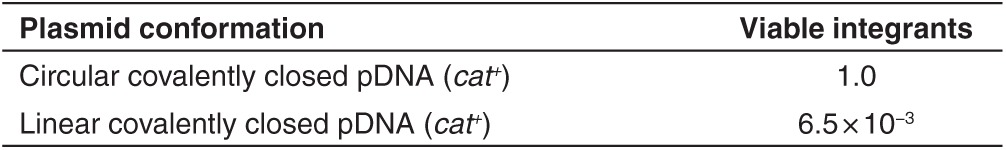
Integration efficiency of linear covalently closed pDNA vectors into human cells
